# Decarburization Control of H13 Steel Under Varying Process Pressures During Austenitization

**DOI:** 10.3390/ma19061272

**Published:** 2026-03-23

**Authors:** Gi-Hoon Kwon, Byoungho Choi, Su-Young Choi, Kyoung Jun An, Kyoung Il Moon

**Affiliations:** Customized Manufacturing R&D Department (Siheung Technology Support Center), Korea Institute of Idustrial Technology, Siheung 15014, Republic of Korea; kgh9900a@kitech.re.kr (G.-H.K.);

**Keywords:** decarburization, carbon diffusion, robin boundary condition, biot number

## Abstract

Decarburization during austenitization degrades the surface integrity and mechanical performance of tool steels, yet the quantitative influence of process pressure remains unclear. In this study, the effect of process pressure on the decarburization behavior of H13 tool steel was investigated. Specimens were austenitized at 920–1020 °C for 60 min under pressures ranging from 0.01 to 760 Torr. Carbon concentration profiles were measured by electron probe microanalysis, and hardness degradation and mass loss were evaluated. A one-dimensional diffusion model with a Robin boundary condition was applied to describe the coupled effects of carbon diffusion and surface reaction. High-vacuum conditions suppressed decarburization, whereas increasing pressure accelerated carbon loss, leading to deeper decarburized layers and pronounced hardness reduction. The model reproduced the experimental results and revealed a pressure-dependent transition in the dominant decarburization mechanism.

## 1. Introduction

Heat treatment is essential for controlling the microstructure and mechanical properties of tool steels. Among alloying elements, carbon plays a decisive role during austenitization and subsequent quenching. The carbon content in austenite determines hardenability and strongly influences martensitic transformation behavior. It also affects lattice distortion in martensite and therefore directly contributes to hardness and strength [1,2,3,4]. Precise control of carbon distribution is therefore critical for maintaining surface integrity and dimensional stability in hot-work tool steels such as H13.

During austenitization, surface decarburization may occur when carbon reacts with residual oxygen or moisture in the furnace atmosphere. Carbon depletion near the surface reduces hardness, shortens wear life and increases the risk of surface cracking [5,6]. Even in vacuum heat treatment, incomplete evacuation leakage or pressure fluctuation may promote decarburization. In industrial practice, the total pressure inside the furnace often varies during heating holding and gas exchange steps. These variations may significantly influence surface reaction kinetics.

Decarburization is generally described as a diffusion-controlled process governed by Fick’s second law [7,8,9]. The rate increases with temperature due to enhanced carbon diffusivity. Surface reactions between carbon and oxidizing species generate CO or CO_2_, which maintain a concentration gradient that drives outward carbon diffusion. Previous studies reported temperature-dependent decarburization behavior in various alloy steels [10]. In highly alloyed steels such as H13, strong carbide-forming elements reduce effective carbon diffusivity and alter transformation behavior [11]. However, prolonged exposure at high temperature still results in significant carbon loss especially near the surface region where carbon gradients are steep.

Several studies investigated the influence of atmosphere on decarburization in H13 steel. Ramezani et al. reported that vacuum heat treatment produced shallower decarburized layers than air atmospheres [12]. Arain et al. compared atmospheric and vacuum heat treatments, focusing mainly on toughness behavior [13]. These studies provided valuable insight into atmospheric effects, but most compared only air and vacuum conditions or examined a single pressure level. Quantitative evaluation of decarburization sensitivity across a broad pressure range remains limited. In addition, few studies have attempted to extract surface reaction parameters or identify the transition of the governing mechanism between surface reaction control and diffusion control.

The present study aims to quantitatively evaluate the influence of process pressure on the decarburization behavior of H13 steel during austenitization. Heat treatment was conducted at 920, 970 and 1020 °C under pressures from 0.01 to 760 Torr. Carbon concentration profiles, hardness distribution, mass loss, wear behavior, and phase evolution were systematically analyzed. A diffusion model incorporating a Robin boundary condition was employed to describe the coupled effect of surface reaction and internal diffusion.

Despite previous investigations, a comprehensive analysis of pressure-dependent decarburization kinetics in H13 steel over four orders of magnitude in pressure has not been reported. This study integrates carbon concentration profiling, inverse determination of the surface carbon transfer coefficient, and Biot number analysis to identify the transition of the governing mechanism with increasing pressure. Furthermore, carbon depletion is directly correlated with hardness degradation wear response and phase fraction evolution. These results provide quantitative guidance for pressure control during industrial heat treatment of H13 tool steel.

## 2. Experimental Procedure

### 2.1. Material and Heat Treatment

The material used in this study was H13 hot-work tool steel. Its chemical composition, determined by inductively coupled plasma optical emission spectroscopy (ICP-OES; PerkinElmer, Optima 3000XL, Waltham, MA, USA), was 0.41C–5.2Cr–0.3Mn–1.3Mo–1.1V–1.0Si (wt.%), with Fe as the balance. The specimens were supplied in the annealed condition, with an initial hardness of approximately 252 HV. Rectangular specimens with dimensions of 10 mm × 10 mm × 50 mm were sectioned parallel to the rolling direction using wire electrical discharge machining. Prior to heat treatment, all specimen surfaces were ground and polished up to 2000 grit to ensure a uniform surface condition.

To investigate the effects of process pressure and temperature on decarburization behavior, heat treatment experiments were performed by systematically varying the austenitizing temperature (920–1020 °C) and process pressure (0.01–760 Torr), while all other parameters were kept constant, as summarized in Table 1. The atmosphere during austenitization was ambient air and only the total pressure was controlled.

Austenitization was performed in a cylindrical horizontal quartz furnace with a hot zone measuring 100 mm in diameter and 260 mm in length. High-vacuum conditions were achieved using a turbo-molecular pump in conjunction with an ionization gauge, whereas low-vacuum conditions were controlled using a rotary pump and a Baratron gauge. The specimens were heated to the target austenitizing temperatures at a rate of 12.5 °C/min and subsequently oil-quenched at an average cooling rate of approximately 90 °C/min. The specimen temperature was directly monitored using a thermocouple positioned at a near-surface location approximately 0.5 mm below the specimen surface, and the heating and cooling rates were determined from the recorded temperature–time curves.

### 2.2. Characterization of Decarburization

After heat treatment, the specimens were sectioned perpendicular to the longitudinal direction, and the cross-sections were mirror-polished for subsequent analysis. Carbon concentration profiles from the surface into the interior were measured using an electron probe microanalyzer (EPMA; JEOL, JXA-8530F, Tokyo, Japan). The measurements were conducted at an accelerating voltage of 5 kV and a beam current of 50 nA, with a step size of 10–20 μm from the surface toward the bulk. For each pressure and temperature condition, three independent specimens were prepared. EPMA line scans were performed at three different locations per specimen. The reported carbon concentration profiles represent the average values and the corresponding standard deviation is reflected in the error bars. The carbon concentration profiles were integrated to calculate the total carbon loss per unit area. To validate the reliability of the EPMA-based carbon loss estimation, the calculated values were compared with the mass loss measured using a microbalance with a resolution of 0.01 mg (AND, GR-200, San Jose, CA, USA). The decarburization depth was defined as the distance from the surface to the point where both the microstructure and hardness distribution recovered to levels comparable to those of the bulk material [14,15]. This definition was further corroborated by optical microstructural observations of the decarburized layer using an optical microscope (Huvitz, HRM-300, Anyang-si, Republic of Korea).

### 2.3. Mechanical and Phase Transformation Analysis

To comprehensively evaluate the effects of carbon depletion due to decarburization on microstructural evolution and mechanical properties, mechanical testing and phase transformation analyses were performed. Mechanical properties were assessed using micro-Vickers hardness testing and tribological measurements. Hardness profiles from the surface toward the interior were measured using a micro-Vickers hardness tester (Mitutoyo, HM-210B, Kawasaki, Japan). For each depth position, seven independent hardness measurements were performed. The plotted values correspond to the average hardness and the error bars represent the standard deviation. Tribological behavior was evaluated using a ball-on-disk tribometer (J&L Tech, JLTB-02, Seongnam-si, Republic of Korea) equipped with an SUJ2 steel ball under a normal load of 10 N, a sliding speed of 0.1 m/s, and a total sliding distance of 1000 m. These tests were employed as a supplementary indicator to examine the influence of decarburization-induced surface degradation on wear behavior.

Phase transformation behavior was analyzed to interpret microstructural differences between decarburized and bulk regions. Continuous cooling transformation (CCT) diagrams and phase transformation tendencies were calculated using JMatPro v12, with the carbon concentrations measured by EPMA in the decarburized and bulk regions used as input parameters. The predicted phase transformation behavior was validated by electron backscatter diffraction (EBSD; EDAX, Velocity Super, Paoli, PA, USA) analysis. Rather than focusing on absolute phase fractions, the analysis emphasized relative trends in phase evolution, thereby experimentally elucidating the influence of decarburization-induced carbon depletion on martensite formation behavior and ferrite phase fraction.

### 2.4. Modeling of Decarburization Behavior

In this study, the decarburization behavior occurring during austenitization was assumed to be governed by the combined effects of carbon diffusion within the material and carbon removal reactions at the surface. Accordingly, a one-dimensional diffusion model was employed. The temporal evolution of the carbon concentration, C(x,t), is described by Fick’s second law [2].

(1) $$\frac{\partial C}{\partial t}=D\frac{\partial^{2}C}{\partial x^{2}}$$

Carbon diffusion in austenite was described using a temperature-dependent diffusivity with carbon content dependence based on the empirical relation proposed by Lee et al. [16]:

(2) $$D\left(T,C\right)=exp\left(0.146-0.036C\right)\times exp\left(-\frac{144.3-15.0C+0.37C^{2}}{R_{KJ}T}\right)$$

The nominal carbon content of H13 steel (0.41 wt.%) was used, and the same diffusivity was applied to all conditions because the alloy composition was fixed during the experiments.

Here, C denotes the carbon concentration (wt.%), t represents time, x is the distance measured from the surface, and D is the carbon diffusion coefficient. The initial condition was defined by assuming a uniform bulk carbon concentration throughout the material at the beginning of the heat treatment, as expressed below.

(3) $$C\left(x,0\right)=C_{0}$$

At the surface (x = 0), decarburization occurs as a result of reactions with the surrounding atmosphere. Therefore, a Robin boundary condition was applied to account for the combined effects of surface reaction kinetics and internal carbon diffusion.

(4) $$-D{\frac{\partial C}{\partial x}|}_{x=0}=h\left(C_{s}-C_{g}\right)$$

Here, C_s_ denotes the surface carbon concentration, C_g_ represents the effective carbon concentration in equilibrium with the surrounding atmosphere, and h is the surface carbon transfer coefficient. The Robin boundary condition enables realistic representation of decarburization by simultaneously accounting for surface reaction kinetics and internal carbon diffusion; it is therefore well-suited for comparing decarburization behavior under different process pressures. To quantitatively evaluate the relative contributions of surface reaction-controlled and diffusion-controlled mechanisms, the Biot number was defined as follows [17,18].

(5) $$Bi=\frac{hL}{D}$$

Here, L represents the characteristic length, defined as the effective distance over which decarburization proceeds. In general, larger Biot numbers indicate diffusion-controlled behavior dominated by internal carbon diffusion, whereas smaller Biot numbers signify surface reaction-controlled behavior. The surface carbon transfer coefficient, h, was determined through inverse analysis by minimizing the deviation between the carbon concentration profiles measured by EPMA and those predicted by the diffusion model. The obtained values of h and Bi were subsequently employed to predict decarburization behavior over the investigated pressure range.

## 3. Results

### 3.1. Carbon Concentration Profiles and Decarburization Depth

**Figure 1:**
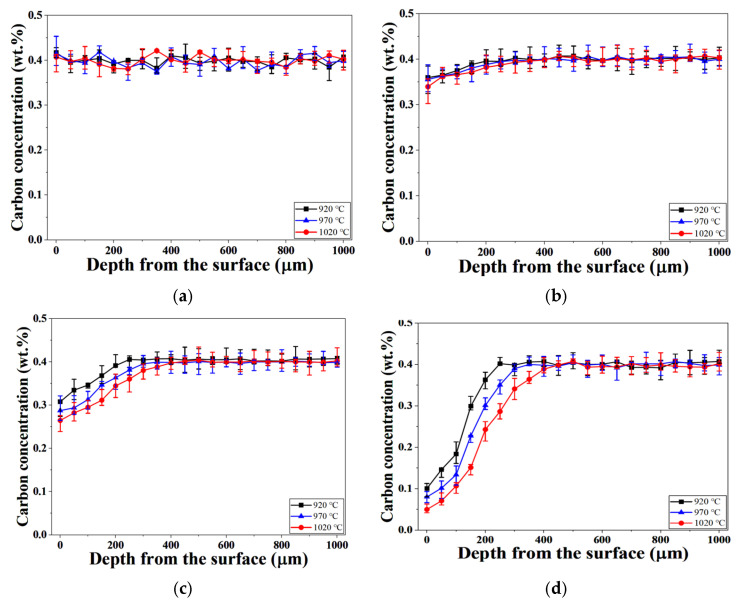
Carbon concentration profiles according to depth from the surface for H13 steel austenitized under different process pressures: (**a**) 0.01 Torr; (**b**) 10 Torr; (**c**) 100 Torr; (**d**) 760 Torr.

Figure 1 presents the carbon concentration profiles as a function of depth from the surface in H13 steel austenitized under different process pressures. Under high-vacuum conditions (0.01 Torr), the carbon concentration remained nearly uniform from the surface to the interior at all austenitizing temperatures, indicating effective suppression of decarburization.

In contrast, with increasing process pressure, a progressive decrease in carbon concentration was observed near the surface, followed by gradual recovery toward the bulk carbon level with increasing depth. This behavior became increasingly pronounced at pressures of 100 Torr and 760 Torr. At a given pressure, higher austenitizing temperatures resulted in more severe surface carbon depletion and greater decarburization depth, reflecting the enhanced carbon diffusion kinetics at elevated temperatures.

**Figure 2:**
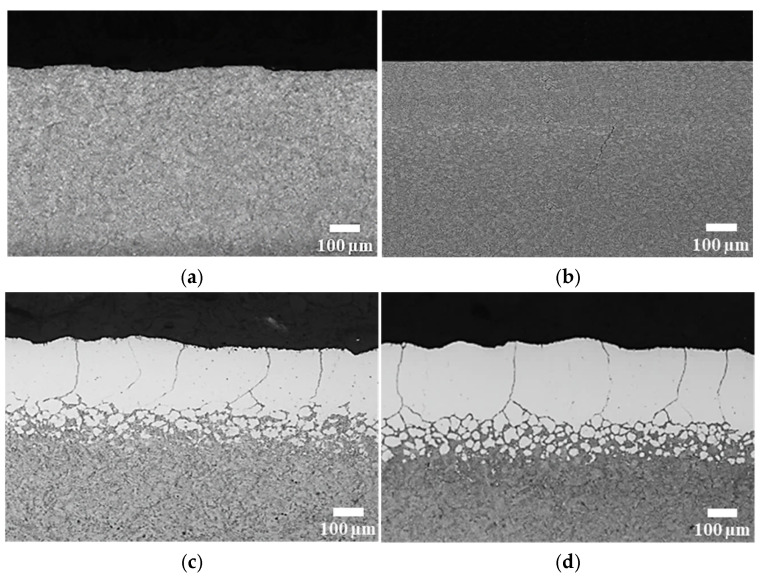
Optical micrographs of H13 steel austenitized at 1020 °C under different process pressures: (**a**) 0.01 Torr; (**b**) 10 Torr; (**c**) 100 Torr; (**d**) 760 Torr.

Figure 2 shows optical micrographs of cross-sections of H13 steel austenitized under different process pressures. At 0.01 Torr, no clear microstructural difference was observed between the surface and the interior. As the process pressure increased, however, a distinct light-etching layer gradually developed near the surface. This region is interpreted as a decarburized layer dominated by ferrite, formed as a result of significant carbon depletion [19,20].

At pressures of 100 Torr and 760 Torr, a continuous decarburized layer extending several hundred micrometers from the surface was clearly observed. These microstructural features are in good agreement with the carbon concentration profiles measured by EPMA and the corresponding trends in decarburization depth.

**Figure 3:**
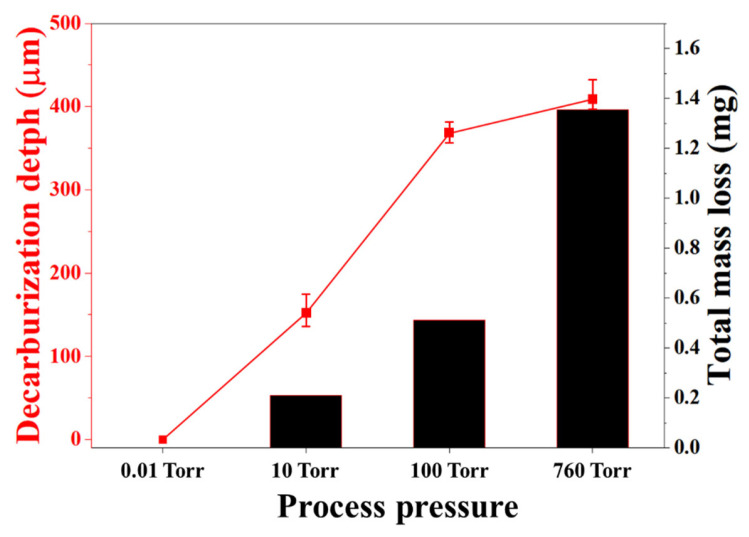
Decarburization depth and total mass loss of H13 steel as a function of process pressure.

Figure 3 compares the decarburization depth estimated from EPMA-measured carbon concentration profiles with the total mass loss measured using a microbalance as a function of process pressure. Under the 0.01 Torr condition, decarburization was negligible, resulting in minimal decarburization depth and mass loss. At 10 Torr, a decarburized layer approximately 150 μm thick formed, accompanied by a mass loss of about 0.2 mg.

With increasing pressure, decarburization intensified markedly. At 100 Torr, the decarburization depth increased to approximately 370 μm, with a corresponding mass loss of around 0.5 mg. Under atmospheric pressure (760 Torr), the decarburized layer extended beyond 400 μm, and the total mass loss reached approximately 1.3 mg. The close agreement between the decarburization depth derived from EPMA measurements and the independently measured mass loss demonstrates the quantitative reliability of the carbon concentration analysis.

### 3.2. Validation of the Diffusion Model and Pressure-Dependent Surface Kinetics

**Figure 4:**
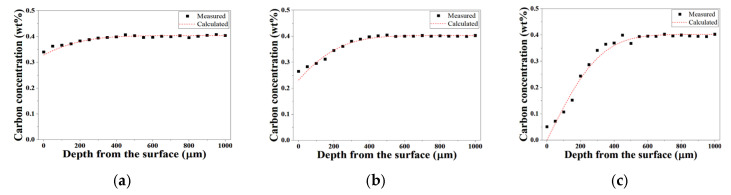
Comparison between EPMA-measured carbon concentration profiles and fitted diffusion model predictions at 1020 °C under different process pressures: (**a**) 10 Torr; (**b**) 100 Torr; (**c**) 760 Torr.

Figure 4 compares the EPMA-measured carbon concentration profiles and the corresponding diffusion model predictions at 1020 °C for 60 min. As shown in Figure 4a–c, the pressure conditions correspond to 10 Torr, 100 Torr, and 760 Torr, respectively. The model simultaneously accounts for one-dimensional carbon diffusion in austenite and carbon removal reactions at the specimen surface.

For all pressure conditions, the predicted profiles exhibit excellent agreement with the experimental data. In the modeling, carbon reaching the surface was assumed to be continuously removed to the gas phase under decarburizing conditions. Accordingly, the equilibrium surface carbon concentration (C_eq_) was set to zero. Since austenitization was conducted in ambient air with controlled total pressure, surface oxidation inevitably accompanied decarburization. In addition, residual oxygen present in the furnace atmosphere can further contribute to decarburization reactions by forming CO or CO_2_ at the steel surface. The effect becomes more significant as the process pressure increases, since the partial pressure of oxidizing species also increases. Increasing pressure enhances the availability of oxidizing species (e.g., O_2_ and H_2_O) at the surface, which promotes carbon removal reactions and increases the apparent surface reaction rate.

The surface carbon transfer coefficient (h) for each pressure condition was determined by inverse analysis through minimization of the deviation between measured and calculated profiles. As a result, high coefficients of determination were obtained (R^2^ = 0.97, 0.99, and 0.96 for 10 Torr, 100 Torr, and 760 Torr, respectively), demonstrating that the coupled diffusion–surface reaction model reliably reproduces decarburization behavior over a wide range of process pressures.

**Figure 5:**
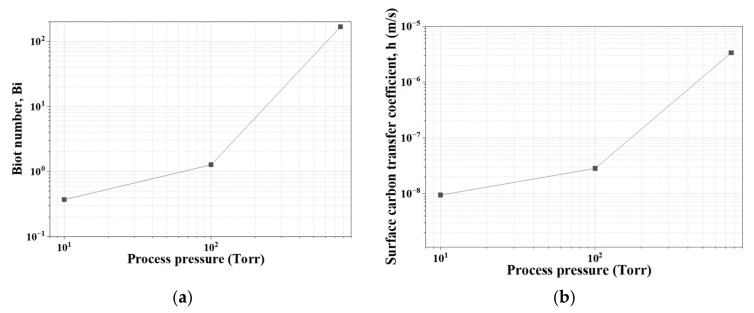
Variation in (**a**) surface carbon transfer coefficient h and (**b**) Biot number Bi as a function of process pressure at 1020 °C.

Figure 5 summarizes the variation in the surface carbon transfer coefficient h and the corresponding Biot number as a function of process pressure. The surface carbon transfer coefficients determined by inverse analysis were 9.43 × 10^−9^ m/s at 10 Torr, 2.83 × 10^−8^ m/s at 100 Torr, and 3.34 × 10^−6^ m/s at 760 Torr. These values increase markedly with pressure, indicating the progressive enhancement of surface carbon removal kinetics under higher oxidizing potential.

The obtained h values fall within the range reported in previous decarburization studies under oxidizing or low-vacuum conditions, which typically span 10^−8^–10^−4^ m/s depending on temperature and oxygen partial pressure [21]. Although the exact magnitude depends on alloy composition and atmosphere control, the order of magnitude and the increasing trend with pressure are consistent with the literature data.

Using these values, the Biot number was calculated to evaluate the governing mechanism. Bi was smaller than unity at 10 Torr, approached unity at 100 Torr, and became much greater than unity at 760 Torr, indicating a transition from mixed surface reaction–diffusion control to diffusion-controlled decarburization. This result demonstrates that process pressure directly governs not only the extent of decarburization but also the dominant kinetic regime.

**Figure 6:**
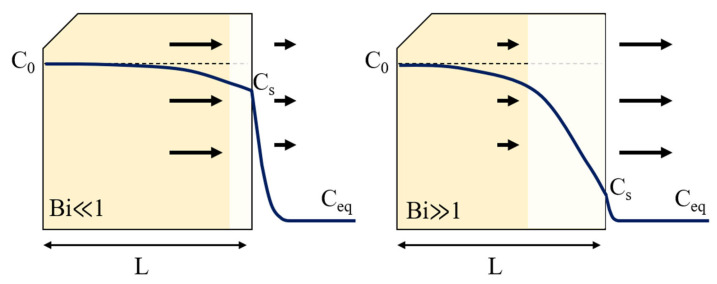
Schematic illustration of decarburization behavior expressed in terms of carbon concentration profiles under different Biot number regimes.

Figure 6 schematically illustrates the decarburization behavior in terms of carbon concentration profiles under different Biot number regimes. When Bi ≪ 1, surface reaction is slow relative to internal carbon diffusion, resulting in a mild concentration gradient and limited decarburization. For intermediate Bi values (Bi ≈ 1), a mixed control regime is expected, where both surface reaction and diffusion contribute to the kinetics [22]. In contrast, when Bi ≫ 1, rapid surface carbon removal maintains a low surface carbon concentration (Cₛ ≈ C_eq_), leading to a steep concentration gradient and diffusion-controlled decarburization. This interpretation is consistent with the pressure-dependent increase in the surface carbon transfer coefficient (h) obtained from the diffusion model.

In addition to pressure, austenitization temperature also influences decarburization through the temperature dependence of carbon diffusivity. As shown in Figure 1, higher temperatures produce deeper carbon depletion near the surface. Therefore, decarburization during austenitization is governed by the coupled effects of temperature-dependent diffusion and pressure-dependent surface reaction kinetics.

### 3.3. Hardness Degradation and Its Correlation with Carbon Depletion

**Figure 7:**
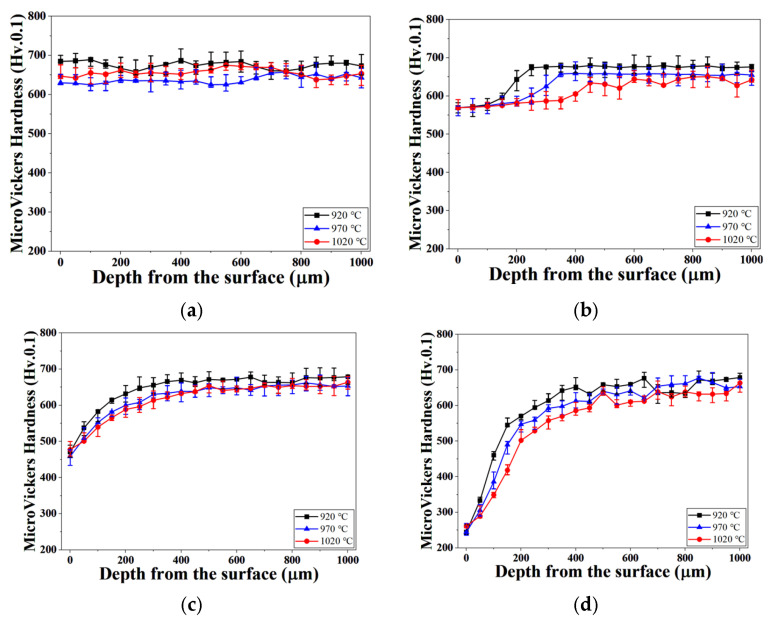
Micro-Vickers hardness profiles from the surface of heat-treated specimens as a function of process pressure at an austenitizing temperature of 1020 °C: (a) 0.01 Torr; (b) 10 Torr; (c) 100 Torr; (d) 760 Torr.

Figure 7 presents the micro-Vickers hardness profiles measured from the surface toward the interior of H13 steel specimens austenitized at 1020 °C under different process pressures. Under the 0.01 Torr condition (Figure 7a), hardness remained nearly constant from the surface to the bulk, indicating effective suppression of decarburization, consistent with the negligible surface carbon loss observed in the carbon concentration profiles. At 10 Torr (Figure 7b), a reduced hardness region was observed near the surface, followed by gradual recovery with increasing depth, indicating the formation of a partially decarburized layer governed by mixed surface reaction and diffusion control. When the pressure increased to 100 Torr (Figure 7c), a pronounced low-hardness region extending several hundred micrometers from the surface was observed, reflecting intensified decarburization under higher pressure conditions.

Under atmospheric pressure (Figure 7d), a steep hardness drop occurred near the surface, and the low-hardness zone extended deep into the interior. This behavior corresponds to the formation of a thick decarburized layer under conditions dominated by rapid surface carbon removal (Bi ≫ 1). Overall, the hardness profiles clearly demonstrate that increasing process pressure during austenitization significantly degrades surface and near-surface mechanical properties.

**Figure 8:**
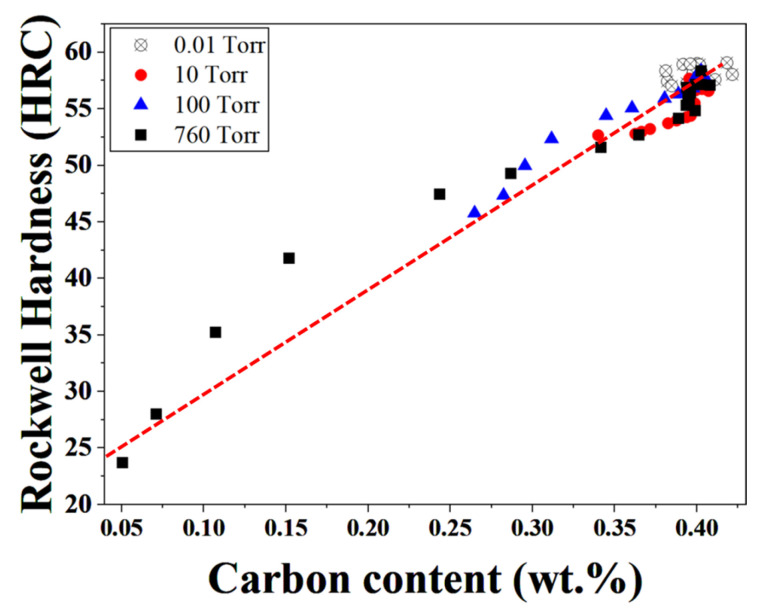
Relationship between carbon content and Rockwell hardness (HRC) of H13 steel austenitized at 1020 °C under different process pressures.

Figure 8 shows the relationship between carbon content and hardness for H13 steel austenitized at 1020 °C under different process pressures. Regardless of pressure condition or measurement location, all data exhibit a clear linear increase in hardness with increasing carbon content.

Regression analysis indicates that the hardness can be well described by the linear relationship (HRC = 81.73 C (wt.%) + 24.57), demonstrating that carbon content is the primary factor governing hardness degradation in the decarburized surface and near-surface regions. Notably, the data obtained under different process pressures collapse onto a single regression line, indicating that the observed hardness reduction is controlled by carbon depletion rather than by the process pressure itself. These results confirm that the mechanical property degradation induced by decarburization can be quantitatively explained by carbon loss prior to microstructural transformation effects, thereby providing a direct link between decarburization behavior and surface mechanical performance.

### 3.4. Tribological Response and Its Correlation with Decarburization Behavior

**Figure 9:**
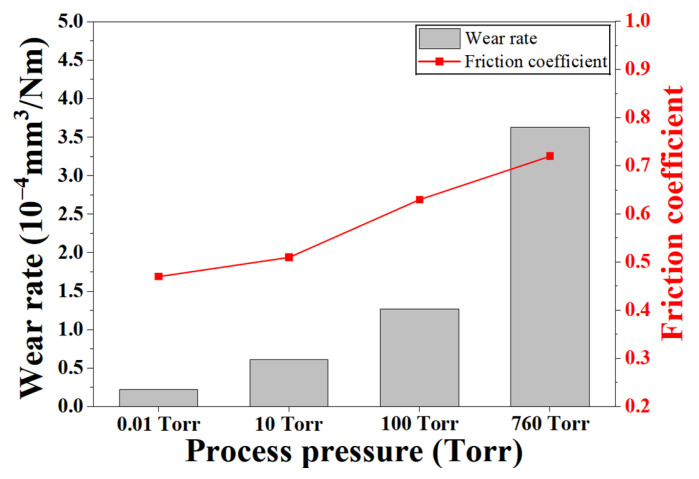
Variation in wear rate and friction coefficient of H13 steel austenitized at 1020 °C as a function of process pressure.

Figure 9 shows the variation in wear rate and friction coefficient of H13 steel austenitized at 1020 °C as a function of process pressure. The wear rate increased monotonically with increasing pressure, reaching its maximum under atmospheric pressure (760 Torr). This trend directly reflects the reduction in surface and near-surface hardness observed in Figure 7.

The friction coefficient also increased progressively with increasing process pressure. Under high-vacuum conditions (0.01 Torr), a low and stable friction coefficient was observed. In contrast, higher pressures of 100 Torr and 760 Torr resulted in increased friction coefficients of approximately 0.65 and 0.72, respectively. This behavior can be attributed to the formation of a low-hardness surface layer caused by decarburization, which is more susceptible to plastic deformation and adhesive wear during sliding [23,24]. These results demonstrate that the quantitative carbon content–hardness relationship established in Figure 8 is directly manifested in tribological performance. Accordingly, controlling the atmospheric pressure during austenitization is essential not only for maintaining surface hardness but also for improving the wear resistance and frictional performance of H13 tool steel.

### 3.5. Influence of Carbon Depletion on Phase Transformation Behavior

**Figure 10:**
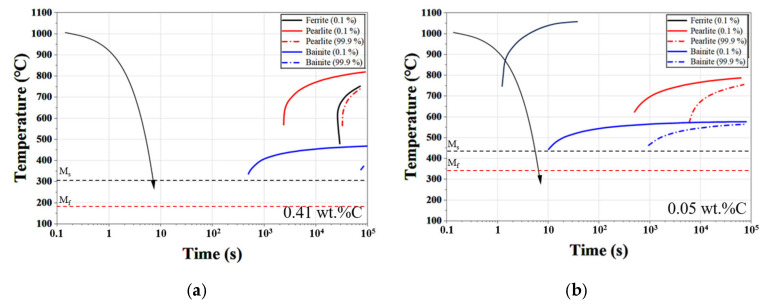
Comparison of continuous cooling transformation (CCT) diagrams calculated for (**a**) bulk carbon composition (0.41 wt.%C) and (**b**) severely decarburized surface composition (0.05 wt.%C), illustrating the effect of carbon depletion on phase transformation behavior.

Figure 10 compares the continuous cooling transformation (CCT) behavior under identical cooling conditions for different carbon concentrations. As the carbon content decreases, the start temperature (Mₛ) of martensite increases, while the martensitic transformation region becomes narrower. Concurrently, the bainite and pearlite transformation regions shift toward higher temperatures and longer transformation times.

These changes indicate that even under the same cooling rate, transformation behavior can differ significantly in surface regions where carbon content has been reduced by decarburization. In the present study, carbon depletion was experimentally shown to directly affect hardness and tribological properties. Accordingly, the CCT curves presented in Figure 10 are provided as a reference to illustrate that changes in carbon concentration may also influence phase transformation behavior. Such transformation-related effects may further amplify the degradation of surface properties, in addition to the direct effect of carbon depletion.

**Figure 11:**
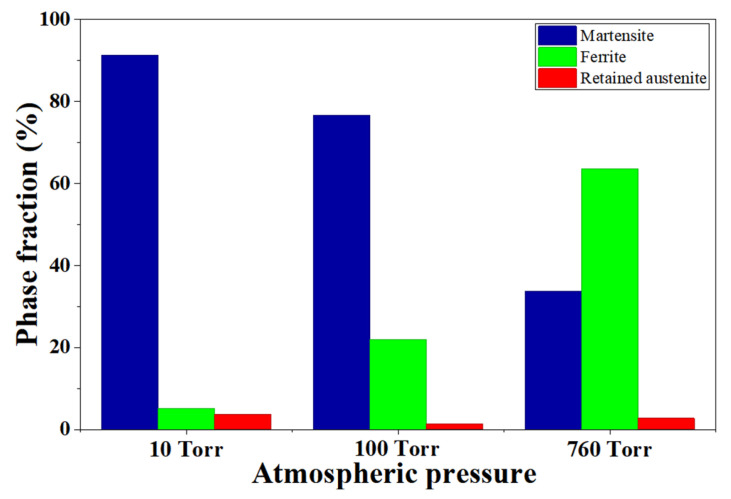
EBSD-based phase fraction evolution near the surface of H13 tool steel as a function of atmospheric pressure during austenitization at 1020 °C.

Figure 11 shows the EBSD phase distribution and the phase fraction evolution near the surface of H13 tool steel austenitized at 1020 °C under different pressures. Quantitative EBSD analysis indicates that the martensite fraction decreased from 91.4% at 10 Torr to 34.1% at 760 Torr, while the ferrite fraction increased from 5.2% to 63.2% as shown in Table 2. The retained austenite fraction remained low under all conditions.

At low pressure (10 Torr), martensite was the dominant phase near the surface. As pressure increased, the ferrite fraction increased while the martensite fraction decreased. This phase evolution results from enhanced decarburization at higher pressures, which reduces the carbon concentration in the near-surface region. The trend agrees with the CCT predictions shown in Figure 10, where reduced carbon content suppresses martensitic transformation and promotes ferrite formation. However, even at atmospheric pressure, a fully ferritic structure was not observed because H13 steel contains alloying elements such as Cr, Mo, and V that stabilize martensite even at relatively low carbon levels.

These EBSD results confirm that carbon depletion caused by decarburization changes the near-surface phase distribution and contributes to the reduction in surface hardness and tribological performance discussed earlier.

## 4. Conclusions

1. Increasing process pressure during austenitization significantly enhanced decarburization in H13 tool steel. Surface carbon concentration decreased progressively with pressure, and the decarburization depth increased from negligible levels at 0.01 Torr to more than 400 μm at 760 Torr.
2. The carbon concentration profiles measured by EPMA were well-reproduced using a one-dimensional diffusion model with a Robin boundary condition. The fitted surface carbon transfer coefficients were 9.43 × 10^−9^ m/s at 10 Torr, 2.83 × 10^−8^ m/s at 100 Torr, and 3.34 × 10^−6^ m/s at 760 Torr.
3. The calculated Biot number increased with process pressure. At low pressure, Bi was smaller than unity, indicating mixed surface reaction and diffusion control. At atmospheric pressure, Bi became much greater than unity, indicating diffusion-controlled decarburization.
4. Surface carbon depletion led to a corresponding reduction in hardness near the surface. The hardness profiles showed recovery with increasing depth consistent with the carbon concentration distribution. A linear relationship between carbon content and hardness was confirmed.
5. Wear rate and friction coefficient increased with increasing process pressure, reflecting the reduction in near-surface hardness. EBSD analysis showed an increase in ferrite fraction and a decrease in martensite fraction in decarburized regions, consistent with the reduced carbon concentration.

## Figures and Tables

**Table 1:** Summary of heat treatment parameters and experimental conditions used for investigating the decarburization behavior of H13 steel during austenitization.

Parameter	Condition
Material	H13 tool steel (0.41C–5.2Cr–1.3Mo–1.1V–1.0Si, wt.%)
Austenitizing temperature (°C)	920, 970, 1020
Process pressure (Torr)	0.01, 10, 100, 760
Holding time (min)	60
Heating rate (°C/min)	12.5
Furnace type	Horizontal quartz vacuum furnace
Cooling method	Oil quenching
Average cooling rate (°C/min)	90
Specimen size (mm)	10 mm × 10 mm × 50 mm

**Table 2:** Phase fraction determined from EBSD analysis.

Pressure	Martensite (%)	Ferrite (%)	Retained Austenite (%)
10 Torr	91.4	5.2	3.4
100 Torr	76.3	22.0	1.7
760 Torr	34.1	63.2	2.7

## Data Availability

The original contributions presented in this study are included in the article. Further inquiries can be directed to the corresponding author.
